# Multiparametric functional MRI and ^18^F-FDG-PET for survival prediction in patients with head and neck squamous cell carcinoma treated with (chemo)radiation

**DOI:** 10.1007/s00330-020-07163-3

**Published:** 2020-08-26

**Authors:** Roland M. Martens, Thomas Koopman, Cristina Lavini, Meedie Ali, Carel F. W. Peeters, Daniel P. Noij, Gerben Zwezerijnen, J. Tim Marcus, Marije R. Vergeer, C. René Leemans, Remco de Bree, Pim de Graaf, Ronald Boellaard, Jonas A. Castelijns

**Affiliations:** 1 Department of Radiology and Nuclear Medicine, Amsterdam UMC, De Boelelaan 1117, Amsterdam, The Netherlands; 2 Department of Radiology and Nuclear Medicine, Amsterdam UMC, Meibergdreef 9, Amsterdam, The Netherlands; 3 Department of Epidemiology and Biostatistics, Amsterdam UMC, De Boelelaan 1117, Amsterdam, The Netherlands; 4 Department of Radiation Oncology, Amsterdam UMC, De Boelelaan 1117, Amsterdam, The Netherlands; 5 Department of Otolaryngology - Head and Neck Surgery, Amsterdam UMC, De Boelelaan 1117, Amsterdam, The Netherlands; 6 Department of Head and Neck Surgical Oncology, University Medical Center Utrecht, Heidelberglaan 100, Utrecht, The Netherlands

**Keywords:** Squamous cell carcinoma of head and neck, Diffusion magnetic resonance imaging, Magnetic resonance imaging, Positron emission tomography computed tomography, Survival analysis

## Abstract

**Objectives:**

To assess (I) correlations between diffusion-weighted (DWI), intravoxel incoherent motion (IVIM), dynamic contrast-enhanced (DCE) MRI, and ^18^F-FDG-PET/CT imaging parameters capturing tumor characteristics and (II) their predictive value of locoregional recurrence-free survival (LRFS) and overall survival (OS) in patients with head and neck squamous cell carcinoma (HNSCC) treated with (chemo)radiotherapy.

**Methods:**

Between 2014 and 2018, patients with histopathologically proven HNSCC, planned for curative (chemo) radiotherapy, were prospectively included. Pretreatment clinical, anatomical, and functional imaging parameters (obtained by DWI/IVIM, DCE-MRI, and ^18^F-FDG-PET/CT) were extracted for primary tumors (PT) and lymph node metastases. Correlations and differences between parameters were assessed. The predictive value of LRFS and OS was assessed, performing univariable, multivariable Cox and CoxBoost regression analyses.

**Results:**

In total, 70 patients were included. Significant correlations between ^18^F-FDG-PET parameters and DWI-/DCE volume parameters were found (*r* > 0.442, *p* < 0.002). The combination of HPV (HR = 0.903), intoxications (HR = 1.065), PT ADC_GTV_ (HR = 1.252), K^trans^ (HR = 1.223), and V_e_ (HR = 1.215) was predictive for LRFS (C-index = 0.546; *p* = 0.023). N-stage (HR = 1.058), HPV positivity (HR = 0.886), hypopharyngeal tumor location (HR = 1.111), ADC_GTV_ (HR = 1.102), ADC_mean_ (HR = 1.137), D* (HR = 0.862), K^trans^ (HR = 1.106), V_e_ (HR = 1.195), SUV_max_ (HR = 1.094), and TLG (HR = 1.433) were predictive for OS (C-index = 0.664; *p* = 0.046).

**Conclusions:**

Functional imaging parameters, performing DWI/IVIM, DCE-MRI, and ^18^F-FDG-PET/CT, yielded complementary value in capturing tumor characteristics. More specific, intoxications, HPV-negative status, large tumor volume-related parameters, high permeability (K^trans^), and high extravascular extracellular space (V_e_) parameters were predictive for adverse locoregional recurrence-free survival and adverse overall survival. Low cellularity (high ADC) and high metabolism (high SUV) were additionally predictive for decreased overall survival. These different predictive factors added to estimated locoregional and overall survival.

**Key Points:**

*• Parameters of DWI/IVIM, DCE-MRI, and 18F-FDG-PET/CT were able to capture complementary tumor characteristics.*

*• Multivariable analysis revealed that intoxications, HPV negativity, large tumor volume and high vascular permeability (K*^*trans*^*), and extravascular extracellular space (Ve) were complementary predictive for locoregional recurrence.*

*• In addition to predictive parameters for locoregional recurrence, also high cellularity (low ADC) and high metabolism (high SUV) were complementary predictive for overall survival.*

**Electronic supplementary material:**

The online version of this article (10.1007/s00330-020-07163-3) contains supplementary material, which is available to authorized users.

## Introduction

In patients with advanced stage head and neck squamous cell carcinoma (HNSCC), (chemo)radiation is the standard organ-sparing treatment; however, there is still a 50% (35–65%) recurrence rate [1]. In addition to clinical and histological parameters, other sophisticated biomarkers are needed to stratify patients for optimal therapy (e.g., de-escalation, escalation, or switching to surgery) [2, 3]. Being able to correctly identify patients with a favorable prognosis might allow treatment adaptation to reduce long-term toxicity without compromising outcome [4].

Functional imaging techniques capture a variety of biological characteristics, such as cellularity, perfusion, permeability, and glucose metabolism.

Tissue microstructures (i.e., cellularity, necrosis, stroma, hemorrhage) can be assessed by diffusion-weighted MRI (DWI) and quantified by the apparent diffusion coefficient (ADC). An extension of DWI is the intravoxel incoherent motion (IVIM), which can assess both diffusion and perfusion fraction, without contrast injection [5–7].

Perfusion and vessel permeability can be assessed by dynamic contrast-enhanced (DCE) MRI and quantified by the K^trans^ (transfer rate of contrast agent from plasma to extravascular, extracellular space), V_e_ (fractional volume of extracellular extravascular space), and K_ep_ (contrast agent transfer rate from extravascular, extracellular space to plasma) [8].

Glucose metabolism can be assessed by ^18^F-fluoro-deoxy-glucose (^18^F-FDG) positron emission tomography (PET) and is quantified by standardized uptake values (SUV) [9].

Combining modalities might improve predictive accuracy by capturing a variety of tumor characteristics in order to improve predictive accuracy. This could have clinical implications such as guidance for treatment planning, early treatment response, and outcome prediction [7, 10–15]. In contrast, overlapping parameters might be redundant and might reduce protocol efficiency [16]. The predictive values of DWI- and IVIM-MRI, DCE-MRI, and ^18^F-FDG-PET parameters of primary tumor (PT) and lymph node metastasis (LNM) have been only sporadically described in studies, without the use of multivariable Cox regression analysis [14, 17–20] or inclusion of clinical parameters (e.g., HPV status) [15, 21–24].

The aim of our study was to assess (I) the correlations between diffusion-weighted (DWI), intravoxel incoherent motion (IVIM), dynamic contrast-enhanced (DCE) MRI, and 18F-FDG-PET/CT imaging parameters capturing tumor characteristics and (II) their predictive value of locoregional recurrence-free survival (LRFS) and overall survival (OS) in patients with head and neck squamous cell carcinoma (HNSCC) treated with (chemo)radiotherapy.

## Materials and methods

### Patient selection

For this prospective single-center study, approved by our ethical committee (Trial NL3946, NTR4111), written informed consent was obtained from all patients. Previously untreated patients with histologically proven HNSCC, planned for curative (chemo) radiotherapy, and those who underwent ^18^F-FDG-PET/low-dose CT and DWI-DCE-MRI were consecutively included between 2013 and 2018. Exclusion criteria were nasopharyngeal tumors, age < 18 and inadequate image quality. Within 5 weeks after baseline imaging, treatment was initiated consisting of pre-determined radiotherapy (70Gy in 35 fractions in 7 weeks, or accelerated with 70Gy in 35 fractions in 6 weeks) with/without concomitant chemotherapy (3-weekly 100 mg/m^2^ cisplatin), or cetuximab (400 mg/m^2^ before radiotherapy initiation and then weekly 250 mg/m^2^ for 7 weeks). HPV status was determined by p16-immunostaining followed by DNA-PCR on p16-immuno-positive cases. In clinical practice, for lesions outside the oropharynx, HPV positivity is not causally associated with HNSCC [25] and not routinely tested for HPV status, therefore excluded in the survival analyses. Qualitative variables were transformed into numbers: gender (female = 0, male = 1), T-stage (T2 = 2, T3 = 3, T4 = 4), N-stage (N0 = 0, N1 = 1, N2 = 2), HPV (negative = 0, positive = 1), location PT (oropharynx = 1, hypopharynx = 2), smoking (pack years), alcohol (< 3 units/day = 0, ≥ 3 units/day = 1), intoxications (none = 0, smoking < 10 pack years = 0, alcohol < 3 units/day = 0, smoking (> 10 pack years) or alcohol (> 3 units/day) = 1, smoking and alcohol use = 2) [26].

### Imaging

MRI was performed on a 3.0T Ingenuity MR scanner (Philips Healthcare) utilizing a 16-channel neurovascular coil. DWI was performed using fat-suppressed single-shot spin-echo echo-planar imaging (SS-SE-EPI); TR = 500 ms, TE = 105 ms; echo-planar imaging factor = 35; sensitivity encoding factor = 3.5; field of view = 230 × 230 mm; slice thickness = 2 mm; intersection gap = 0.3 mm; matrix = 128 × 128; receiver bandwidth = 2735.7 Hz per pixel. Ten *b* values were used: 0, 10, 25, 50, 75, 150, 300, 500, 750, and 1000 s/mm^2^. The ADC map was produced by vendor-provided software.

DCE-MRI was performed, using 3-dimensional T1-weighted fast field echo (FFE); TR/TE = 4.8/2.4 ms; flip angle = 12; FOV = 230 × 230 × 180 mm; matrix = 144 × 144; 75 dynamic acquisitions of 4.16 s; signal averages = 2. The dynamic scan was preceded by scans with variable flip angles (2, 5, 10, 15, and 20) to estimate quantitative native T1 maps, which were later used to convert signal intensity of the DCE scan into contrast agent concentration curve map [27]. Intravenous bolus injection of 0.2 ml/kg of body weight Gd-DOTA (Dotarem, France) was administered after 3 dynamic acquisitions (3 ml/s followed by 25 ml saline flush).

^18^F-FDG-PET/low-dose CT was performed according to EANM guidelines 2.0 on a Gemini TF-PET/CT (Philips Healthcare) with EARL accreditation [28]. Low-dose CT (120 kV; 30 mAs) was performed. Whole-body ^18^F-FDG-PET/CT was performed in arms down position in radiotherapy mask, from mid-thigh to skull vertex, 60 min after intravenous administration of 2.5 MBq/kg ^18^F-FDG, 2 min per bed position. ^18^F-FDG-PET images were reconstructed using vendor-provided reconstruction protocol with photon attenuation correction, matrix size = 144 × 144, and voxel size = 4 × 4 × 4 mm. Post-reconstruction resolution was 5 mm full width at half maximum.

### Delineation

Whole-lesion delineation was performed manually by two independent observers (J.C. and P.dG., 30 and 15 years of experience in head and neck radiology, respectively) on the ADC map and DCE map. Herewith, T1w, STIR, and T2w maps were used for anatomical correlation, with knowledge of TNM stage and tumor location, but blinded for treatment outcome. Furthermore, the patient largest lymph node metastasis was delineated. DWI/IVIM delineation was performed with VELOCITY software (Varian). To assess the interobserver variability, the correlation (Pearson’s *r*), difference (Wilcoxon signed rank rest), and overlap of delineation (Dice index) were calculated.

^18^F-FDG-PET/CT delineation was performed by semi-automatic delineation by a nuclear medicine specialist (B.Z.) using 50% of tumor-specific SUVpeak threshold, corrected for blood glucose level. Details on this method were published previously [29].

### Feature extraction

Imaging parameters were extracted from both PT and LNM whole-lesion ROIs of each observer. Anatomical total lesion volume, i.e., gross tumor volume (GTV), was calculated for each ROI on each imaging map (ADC_GTV_, DCE_GTV_, and metabolic active tumor volume (MATV)). The following quantitative imaging features were calculated per observer by averaging all voxels included in the whole-lesion ROI.

IVIM feature extraction of perfusion fraction (f), perfusion coefficient (D*), and diffusion coefficient (D) was performed with Olea Sphere (Olea Medical, La Ciotat, France), after motion correction.

DCE-MRI analysis was processed with in-house built software (Dynamo; [27]), performing quantitative pharmacokinetic analysis using the 2-compartment Tofts model [8] with patient-specific arterial input function (AIF) obtained from manual delineating the most cranial part of the external carotid artery. The following features were extracted: K^trans^ (transfer rate of contrast agent from plasma to extravascular, extracellular space); V_e_ (fractional volume of extracellular extravascular space); K_ep_ (transfer rate of contrast agent from extravascular, extracellular space to plasma).

^18^F-FDG-PET/CT in-house built software (Accurate; [28]) automatically calculated SUV_max_, SUV_mean_, and SUV_peak_ (i.e., peak value of 8 highest voxels) based on all included voxels of the ROI, and whole-lesion MATV, and total lesion glycolysis (TLG = SUV_mean_ × MATV).

### Statistical analysis

The average of the above written extracted parameters for both observers was used for analyses.

Correlations were assessed between parameters for PT and LNM separately (Pearson correlation coefficient). Differences in imaging parameters among T-stages, N-stages, and intoxications were assessed with the Kruskal-Wallis tests. In order to capture HPV status-specific tumoral characteristics, associations between parameters of patients with locoregional control (LRC) and failure (i.e., recurrence; LRF), and survival and death (univariate Cox regression analysis) were assessed. Bonferroni’s correction for multiple testing was applied.

Firstly, parameters predicting LRF and death were assessed (univariate Cox regression analysis; significance threshold; *p* < 0.05).

Secondly, multivariable Cox regression analysis was performed of all PT parameters for each modality separately with a backwards Wald test (*p* value significance threshold of 0.157 according to the Tripod statement [30, 31]). All quantitative parameters per modality were corrected for significant clinical parameters (gender, age, T-stage, N-stage, PT location, intoxications) by combination in the backwards Wald elimination analysis.

Thereafter, a CoxBoost analysis was performed to fit a Cox proportional-hazards model by component-wise likelihood-based boosting, to deal with the amount of features relative to the events. Internal validation was performed, using bootstrap cross-validation with 500 bootstrap samples. Due to lacking of LNM parameters in N0 patients, these LNM parameters were excluded in the multimodality CoxBoost analysis in order to remain statistically robust.

All predictive PT parameters were given a score 1 when they were higher than the parameter’s median value, which was based on all included patients. By summing up the points, a risk stratification system was constructed. Thereafter, RFS and OS were assessed, stratified for T-stage, AJCC (7th edition), and risk scores (log-rank test; Kaplan-Meier curves).

## Results

### Patient characteristics

**Fig. 1:**
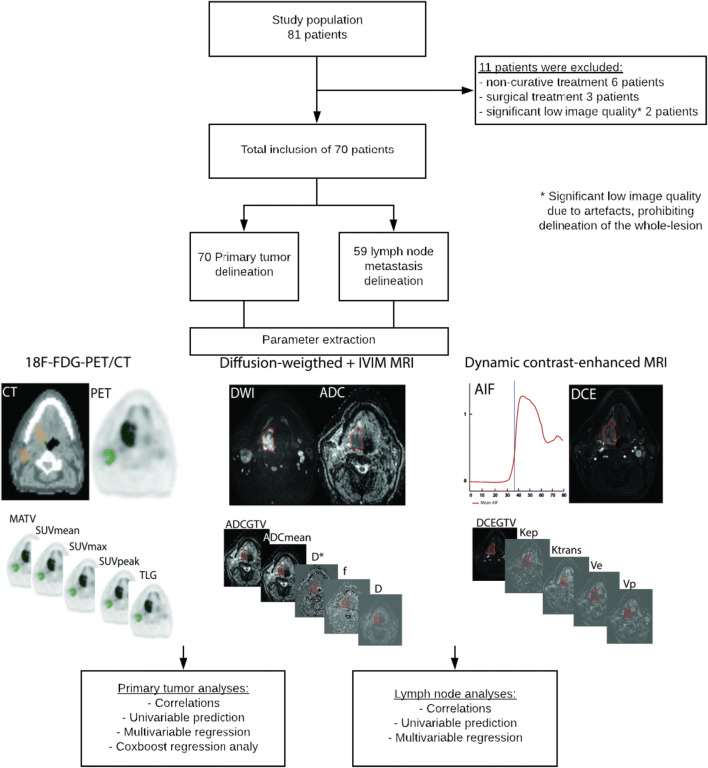
The workflow in our prospective study including the inclusion of eligible patients, delineation of the primary tumor and lymph node metastases of the final included patients, the extraction of quantitative imaging parameters, and metastases predictive assessment of locoregional recurrence-free survival and overall survival using the extracted parameters of the primary tumor and lymph node

Between 2013 and 2018, 81 patients were consecutively recruited (Fig. 1). Nine patients were excluded because of non-curative or surgical treatment and 2 because of significant low image quality.

The final study population consisted of 70 patients (Table 1) with a PT located in the oropharynx (*n* = 56) or hypopharynx (*n* = 14). Among the oropharyngeal tumors, the HPV status of 24 patients was positive (43%). Fifty-four patients received concurrent cisplatin-based chemoradiotherapy. Ten patients received weekly cetuximab with concurrent radiotherapy (70Gy). Six patients received radiotherapy only.

**Table 1:** Baseline characteristics

	Total	HPV+	HPV−
Patients total	70	–	–
Tumor location			
Oropharynx	56	24	32
Hypopharynx	14	2	8*
Gender			
Males	48	16	32
Females	22	10	12
Age, years (IQR)	64 (57.8–69.3)	61.5 (54–67.3)	64.4 (60.3–70)
T-stage			
2	25	16	9
3	17	2	15
4	28	8	20
N-stage			
0	11	2	9
1	15	9	6
2	44	15	29
Daily alcohol (patient amount > 3 units per day)	35	7	28
Smoking (> 20 pack years, mean)	38	7	31
Chemoradiotherapy (Cisplatin)	54	17	37
Radiotherapy with cetuximab	10	4	6
Radiotherapy only	6	3	3
Locoregional recurrence	17	3	14
Death	20	2	18

*HPV was not tested routinely in hypopharyngeal tumors: 2 patients were HPV-positive, 8 patients were HPV-negative, and in 4 patients HPV status was not tested

The mean follow-up was 22.1 months (IQR 14.3–29.4). Seventeen patients (24.3%) developed locoregional recurrence. Twenty (28.6%) patients died during follow-up, all deaths being related to HNSCC (Table 1).

### Associations of imaging parameters per subgroup

Seventy PT ROIs were drawn and 59 lymph node metastasis ROIs (largest LNM) on each modality (Table 1). The comparison of both observers resulted in no significant different values and a high interobserver correlation (Supplement 1). A Dice index in primary tumors of 0.88 at the DWI/IVIM and 0.85 at DCE delineation was found (not tabulated). For LNM, a Dice index of 0.97 at DCE and 0.92 at DWI/IVIM delineation was found (not tabulated). Primary tumor ADC_GTV_, D, f, and D*, DCE_GTV_, and all ^18^F-FDG-PET values (Supplement 2) were significantly higher in advanced T-staged tumors (all *p* ≤ 0.02). In advanced N-staged tumors, PT ADC_GTV_ and V_e_ were significantly higher (*p* = 0.021 and *p* = 0.023, respectively). In HPV-negative tumors, ADC_mean_, D, D*, SUV_max_, SUV_mean_, and SUV_peak_ were significantly higher than HPV-positives (*p* < 0.043). In patients with intoxications, ADC_mean_, D, and D* were significantly different among the different categories (all *p* < 0.027).

In LNM (Supplement 3), K_ep_ and all ^18^F-FDG-PET parameters were significantly higher in advanced N-stages (*p* = 0.025, *p* ≤ 0.016, respectively). In HPV-negative tumors, *D* was found to be significantly higher (*p* = 0.002) and D* lower (*p* = 0.007) than in HPV-positive tumors. In patients with intoxications, *f* was found to be significantly lower (*p* = 0.026).

### Inter-modality correlations

The inter-modality correlation in PT between ^18^F-FDG-PET-, DWI/IVIM-, and DCE-derived parameters (Supplement 4) was only significant among GTV parameters (ADC_GTV_, DCE_GTV_, TLG, and MATV), SUV_peak_, and SUV_mean_ (range: *r* = 0.434–0.915). In LNM (Supplement 5), only volume parameters of LNM ADCGTV, MATV, and DCEGTV correlated significantly (range: *r* = 0.399–725). The intra-modality correlation for PT and LNM (Supplement 6) resulted in significant internal moderate correlation of ADC parameters (range: *r* = 0.432–0.794) and DCE parameters (*r* = 0.637–0.741) and high correlation of ^18^F-FDG-PET parameters (*r* = 0.409–0.995).

### Locoregional recurrence-free survival

The univariate analysis (Table 2) showed that HPV-negative status and the combined intoxications were associated with locoregional recurrence (LRF; *p* = 0.036, *p* = 0.031, respectively). High ADC_GTV_, DCE_GTV_, K^trans^, V_e_, and TLG values of primary tumors were significantly associated with LRF (all *p* ≤ 0.047).

**Table 2:** Univariable and multivariable prediction analysis of locoregional recurrence-free survival

*n* = 70 patient parameters		Local control	Recurrence	Univariable*	Multivariable**	
		Mean ± SD	Mean ± SD	*p* value*	*p* value	HR (95%CI)
Clinical parameters	Gender	0.7 ± 0.5	0.6 ± 0.5	0.665	–	
	Age	62.6 ± 7.9	65.1 ± 6.2	0.167	–	
	T-stage	3.0 ± 0.9	3.1 ± 0.9	0.929	–	
	N-stage	1.4 ± 0.8	1.8 ± 0.4	0.695	–	
	HPV	0.4 ± 0.5	0.2 ± 0.4	0.036	0.036	0.26 (0.08–0.91)
	Location PT	1.2 ± 0.4	1.2 ± 0.44	0.543	–	
	Smoking (PY)	22.8 ± 18.7	30.1 ± 17.3	0.101	–	
	Alcohol (≥ 3drinks/day)	0.43 ± 0.5	0.7 ± 0.5	0.054	–	
	Intoxications					
	None	0.9 ± 0.8	1.4 ± 0.8	0.054	–	
	Smoking or alcohol use	–	–	0.525	–	
	Smoking and alcohol use	–	–	0.031	–	
Primary tumors						
DWI	ADCGTV (cm^3^)	0.8 ± 0.7	1.2 ± 1.4	0.023	0.021	1.69 (1.08–2.64)
	ADC (× 10^3^ mm^2^/s)	1.2 ± 0.2	1.2 ± 0.2	0.166	–	
IVIM	D* (× 10^2^ mm^2^/s)	0.18 ± 0.1	0.18 ± 0.06	0.535	–	
	D (mm^2^/s)	0.96 ± 0.2	1.0 ± 0.2	0.373	–	
	F (× 10^2^ mm^2^/s)	1.4 ± 0.5	1.3 ± 0.6	0.342	–	
DCE	DCE_GTV_ (cm^3^)	11.6 ± 7.8	16.1 ± 14.6	0.047	0.016	1.06 (1.01–1.10)
	K_ep_ (min^−1^)	1.1 ± 0.4	1.2 ± 0.6	0.138	–	
	K^trans^ (min^−1^)	0.6 ± 0.3	0.74 ± 0.3	0.027	0.01	8.50 (1.68–43.1)
	V_e_	1.2 ± 0.8	2.0 ± 1.6	0.008	0.015	1.64 (1.10–2.43)
^18^F-FDG-PET	MATV (cm^3^)	9.7 ± 7.6	13.7 ± 16.5	0.066	0.048	1.04 (1.00–1.09)
	SUV_max_ (Bq)	8.3 ± 3.9	8.7 ± 5.9	0.158	–	
	SUV_mean_ (Bq)	6.0 ± 2.4	6.5 ± 3.8	0.193	–	
	SUV_peak_ (Bq)	7.3 ± 3.2	8.1 ± 5.1	0.133	–	
	TLG (Bq × cm^3^)	66.2 ± 70.3	93.2 ± 104.3	0.039	–	
Lymph node metastases						
DWI	ADC_GTV_ (10^3^ cm^3^)	5.9 ± 4.9	6.1 ± 4.9	0.588	–	
	ADC (× 10^3^ mm2/s)	1.1 ± 0.24	1.2 ± 0.3	0.596	–	
IVIM	D* (mm^2^/s)	2.6 ± 0.7	2.6 ± 0.7	0.914	–	
	D (mm^2^/s)	0.78 ± 0.2	0.8 ± 0.2	0.485	–	
	f (× 10^2^ mm^2^/s)	1.8 ± 0.6	1.76 ± 0.7	0.892	–	
DCE	DCE_GTV_ (cm^3^)	4.7 ± 3.5	6.4 ± 4.7	0.076	0.018	1.18 (1.03–1.36)
	K_ep_ (min^−1^)	0.9 ± 0.6	1.1 ± 0.7	0.692	–	
	K^trans^ (min^−1^)	1.2 ± 1.1	1.4 ± 1.4	0.764	–	
	V_e_	1.3 ± 0.9	1.4 ± 1.5	0.653	–	
^18^F-FDG-PET	MATV (cm^3^)	6.2 ± 7.3	6.4 ± 5.7	0.452	–	
	SUV_max_ (Bq)	8.0 ± 4.4	8.4 ± 3.1	0.554	–	
	SUV_mean_ (Bq)	5.0 ± 2.4	5.4 ± 1.9	0.407	–	
	SUV_peak_ (Bq)	6.1 ± 3.3	6.6 ± 2.6	0.371	–	
	TLG (Bq × cm^3^)	7.6 ± 12.4	7.9 ± 10.3	0.354	–	

*Univariable Cox regression analysis

**Multivariable Cox regression analysis

Univariable and multivariate Cox regression analysis for locoregional recurrence of primary tumor and lymph node metastasis imaging parameters, compared between responders and non-responders. In the multivariate analysis, all parameters per modality were combined, which lead to a loss of intoxications and TLG as remaining predictive parameters for locoregional recurrence

The multivariate analysis per modality (Table 2), corrected for significant clinical parameters (i.e., HPV and intoxications), showed that high primary tumor ADC_GTV_, DCE_GTV_, K^trans^, V_e_, and MATV remained predictive for LRF (all *p* ≤ 0.048). For LNM, only DCE_GTV_ remained significantly predictive for LRF (*p* = 0.018). The subgroup analysis in HPV-negative patients is shown in Supplement 7.

**Fig. 2:**
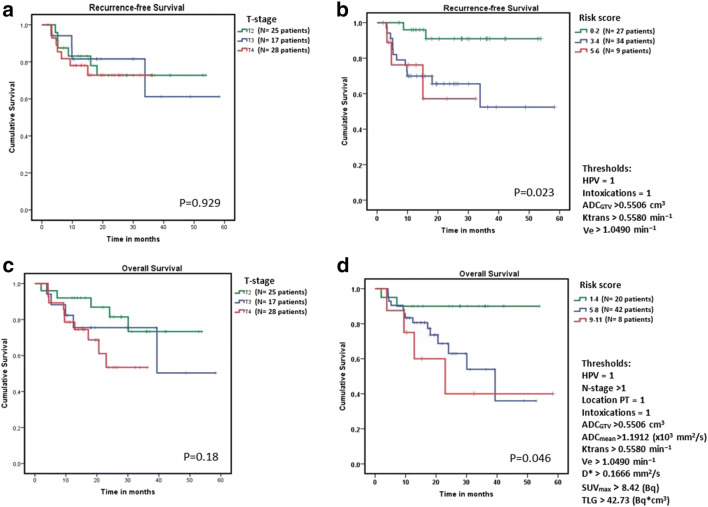
Kaplan-Meier survival curves, which show the recurrence-free survival stratified for (**a**) T-stage and (**b**) for the recurrence risk score. In **a**, the recurrence-free survival is shown, which is not significantly predictive. In **b**, patients were given a risk score by the amount of risk factor points. These risk factors (each with a score of 1 point) were summed up when the predictive quantitative parameters that are higher than the median value of the quantitative parameter or positive clinical parameter (HPV, intoxications, hypopharyngeal PT location or N-stage > 1). The median value of quantitative parameters was calculated based on all included patients. This risk score stratification system is found significantly predictive. In **c**, the overall survival is shown, which is stratified for T-stage, which is not significant predictive. In **d**, the overall survival is shown, stratified for the risk score groups, which is found significantly predictive

The multivariable CoxBoost analysis (Table 4), combining all modalities and clinical parameters, showed that HPV status, intoxications, ADC_GTV_, K^trans^, and V_e_ remained predictive for LRF (C-index of 0.546). The log-rank test (Fig. 2) showed that these risk factors were significantly predictive (*p* = 0.023) for LRF (Fig. 2b), whereas risk stratification per T-stage (Fig. 2a) was not significantly predictive (*p* = 0.92).

### Overall survival

Primary tumor univariate analysis (Table 3) showed that clinical parameters HPV status, PT location, intoxications (*p* ≤ 0.047), and imaging parameters ADC_GTV_, ADC_mean_, D*, D, DCE_GTV_, V_e_, MATV, and SUV_max,_ were significantly associated with OS (all *p* ≤ 0.047). For LNM, SUV_max_, SUV_mean_, and SUV_peak_ were associated with OS (all *p* ≤ 0.015).

**Table 3:** Univariable and multivariable prediction analysis for overall survival

*n* = 70 patient parameters		Survival	Death	Univariable*	Multivariable**	
		Median	Median	*p* value	*p* value	HR (95%CI)
Clinical parameters	Gender	0.6 ± 0.5	0.8 ± 0.4	0.321	–	
	Age	62.4 ± 8.0	65.2 ± 6.0	0.129	–	
	T-stage	3 ± 0.9	3.3 ± 0.9	0.208	–	
	N-stage	1.4 ± 0.8	1.7 ± 0.7	0.214	–	
	HPV	0.5 ± 0.5	0.1 ± 0.3	0.008	0.008	0.139 (0.03–0.60)
	Location PT	1.1 ± 0.3	1.4 ± 0.5	0.047	–	
	Smoking (PY)	22.3 ± 18.9	30.1 ± 16.8	0.066	–	
	Alcohol (≥ 3drinks/day)	0.4 ± 0.5	7 ± 0.5	0.09	–	
	Intoxications	0.9 ± 0.8	1.5 ± 0.8			
	None	–	–	0.029	–	
	Smoking or alcohol use	–	–	0.255	–	
	Smoking and alcohol use	–	–	0.012	–	
Primary tumors						
DWI	ADC_GTV (_× _10_^3^_cm_^3^_)_	0.7 ± 0.7	1.35 ± 1.3	0.004	0.004	1.748 (1.20–2.55)
	ADC_mean_ (× 10^3^ mm^2^/s)	1.2 ± 0.2	1.3 ± 0.2	0.024	–	
IVIM	D* (× 10^2^ mm^2^/s)	0.19 ± 0.07	0.16 ± 0.05	0.032	0.016	< 0.001 (< 0.001–0.1)
	D (mm^2^/s)	0.9 ± 0.2	1.0 ± 0.2	0.009	–	
	f (× 10^2^ mm^2^/s)	1.4 ± 0.5	1.5 ± 0.5	0.786	–	
DCE	DCE_GTV_ (cm^3^)	10.7 ± 10.7	17.5 ± 13.7	0.006	0.001	1.059 (1.02–1.1)
	K_ep_ (min^−1^)	1.1 ± 0.4	1.2 ± 0.5	0.219	–	
	K^trans^ (min^−1^)	0.59 ± 0.3	0.68 ± 0.3	0.089	–	
	V_e_	1.2 ± 0.7	2.0 ± 1.7	0.004	0.019	5.514 (1.32–23.1)
^18^F-FDG-PET	MATV (cm^3^)	8.9 ± 6.7	15.0 ± 15.9	0.003	0.088	1.039 (0.99–1.09)
	SUV_max_ (Bq)	8.6 ± 3.4	11.6 ± 5.8	0.001	0.001	1.189 (1.07–1.32)
	SUV_mean_ (Bq)	5.6 ± 2.1	7.4 ± 3.8	0.001	–	
	SUV_peak_ (Bq)	6.8 ± 2.7	9.2 ± 5.0	0.001	–	
	TLG (Bq × cm^3^)	55.7 ± 56.1	114 ± 111	0.0002	–	
Lymph node metastasis						
DWI	ADCGTV (× 10^3^)	6.0 ± 4.5	5.9 ± 5.2	0.91	–	
	ADC_mean_	1.1 ± 0.06	1.1 ± 0.3	0.757	–	
IVIM	D* (× 10 mm^2^/s)	2.6 ± 0.7	2.6 ± 0.8	0.915	–	
	D (mm^2^/s)	0.8 ± 0.2	0.8 ± 0.2	0.233	–	
	F (× 10^2^ mm^2^/s)	1.8 ± 0.5	1.8 ± 0.6	0.44	–	
DCE	DCE_GTV_ (cm^3^)	5.1 ± 3.9	5.0 ± 4.0	0.895	–	
	K_ep_ (min^−1^)	1.0 ± 0.6	0.9 ± 0.5	0.607	–	
	K^trans^ (min^−1^)	1.2 ± 0.9	1.5 ± 1.6	0.388	–	
	V_e_	1.2 ± 0.7	1.6 ± 1.4	0.131	–	
^18^F-FDG-PET	MATV (cm^3^)	5.8 ± 5.7	7.3 ± 9.1	0.177	–	
	SUV_max_ (Bq)	7.3 ± 3.7	10.0 ± 4.3	0.015	0.055	0.563 (0.43–0.74)
	SUV_mean_ (Bq)	4.6 ± 2.1	6.3 ± 2.3	0.005	0.022	3.536 (2.56–5.26)
	SUV_peak_ (Bq)	5.6 ± 2.8	7.7 ± 4.5	0.009	–	
	TLG (Bq × cm^3^)	7.0 ± 10.0	9.1 ± 15.6	0.143	–	

*Univariate Cox regression analysis

**Multivariable Cox regression per modality

Univariable and multivariable Cox regression analysis of PT and LNM between survivors and patients who died. In the multivariable analysis, all parameters per modality were combined, which lead to a loss of hypopharyngeal PT location, intoxications, ADC_mean_, D, K^trans^, SUVmean, SUV_peak_ and TLG, and LNM SUV_peak_ as remaining predictive parameters for OS

In multivariate analysis per single modality (Table 3), corrected for clinical parameters (i.e., HPV status, hypopharyngeal PT location, intoxications), ADC_GTV_ (*p* = 0.004), D* (*p* = 0.016), DCE_GTV_ (*p* = 0.001), V_e_ (*p* = 0.019), MATV (*p* = 0.088), and SUV_max_ (*p* = 0.001) remained predictive for OS. In LNM, only SUV_max_ (*p* = 0.055, HR = 0.563) and SUV_mean_ (*p* = 0.005, HR 3.536) remained predictive for OS. The subgroup analysis in HPV-negative patients is shown in Supplement 7.

The multivariable CoxBoost analysis combining all PT parameters of all modalities, including all clinical parameters (Table 4), shows that N-stage, HPV status, PT location, intoxications, PT ADC_GTV_, ADC_mean_, D*, K^trans^, V_e_, SUV_max_, and TLG remain predictive for OS, with a C-index of 0.664.

**Table 4:** Multimodality CoxBoost regression analysis

Parameters		Locoregional recurrence-free survival (LRFS) (C-index = 0.546)		Overall survival (OS) (C-index = 0.664)	
		Hazard ratio	95% CI**	Hazard ratio	95% CI**
Clinical	Gender	–		–	
	Age	–		–	
	T-stage	–		–	
	N-stage	–		1.058	0.66–5.14
	HPV-positive status*	0.889	0.08–1.16	0.886	0.07–2.50
	Location (hypopharynx)	–		1.111	1.07–9.70
	Smoking (> 10 PY)	–		–	
	Intoxications (none/smoke-or-alc/both)	1.065	0.83–3.89	1.145	0.93–5.22
DWI	ADC_GTV_	1.293	1.00–2.56	1.102	0.54–4.23
	ADC_mean_	–		1.137	0.07–42.02
IVIM	D*	–		0.862	0.00–56.27
	D	–		–	
	f	–		–	
DCE	DCE_GTV_	–		–	
	K_ep_	–		–	
	K^trans^	1.223	1.09–28.01	1.106	0.79–29.10
	V_e_	1.214	0.90–1.92	1.195	0.76–1.68
^18^F-FDG-PET/CT	MATV	–		–	
	SUV_max_	–		1.094	0.93–1.27
	SUV_mean_	–		–	
	SUV_peak_	–		–	
	TLG	–		1.433	0.99–1.02

*HPV-negative status: HR = 1.1074 for recurrence, HR = 1.1284

**The 95% confidence intervals were calculated from the multivariable analysis

Multivariable CoxBoost regression analysis of primary tumor imaging parameters to predict locoregional recurrence and overall survival. The C-index (taking the area under the curve over time into account) and hazard ratios (HR) are shown. The adverse locoregional recurrence-free survival is predicted significantly by HPV negativity, intoxications, ADC_GTV_, K^trans^, and V_e_. The adverse overall survival predicted significantly by N-stage, HPV negativity, hypopharyngeal tumor location, intoxications, ADC_GTV_, ADC_mean_, D*, K^trans^, V_e_, SUV_max_, and TLG

Predictive parameters scored as risk factors for OS (Fig. 2) were significantly predictive (*p* = 0.046) in the log-rank test (Fig. 2d) when combined, whereas risk stratification per T-stage (Fig. 2c) was found not significant (*p* = 0.188).

## Discussion

In this study, correlations between pretreatment DWI/IVIM, DCE-MRI, and ^18^F-FDG-PET/CT parameters were assessed in order to capture predictive tumor characteristics for LRFS and OS in pharyngeal SCC patients treated with (chemo)radiotherapy.

### Tumor characteristics

Advanced stage tumors (high T-stage) and HPV-negative status had significant higher diffusion (high ADC_mean_, *D*), higher permeability (K^trans^, V_e_), and lower perfusion (low f and D*), implying different tumor characteristics than early stage and HPV-positive tumors. These parameters were also found to be associated with an adverse outcome. This is in line with literature, which described the decrease of cellularity due to apoptosis/necrosis (increased ADC_mean_ and *D*) to be associated with treatment resistance and thereby with poor prognosis [18]. In contrast, in studies which excluded areas of necrosis in the ROI, lower ADC values were found in high-grade tumors with high cellularity. In the current study, HPV-negative patients had a higher ADC value than HPV-positive patients, which was in line with other studies regardless of including [10] or excluding [32–35] necrotic areas. An increase of permeability (increased K^trans^) is possibly due to tumor neoangiogenesis, which increases immature incompetent vessel leakage, thereby increasing the fraction in the extracellular extravascular space (increased V_e_), causing higher interstitial fluid pressure and lower flow [16, 36]. The reduced perfusion (low blood flow and volume; low D* and f, respectively) results in worse access to chemotherapeutic drugs and oxygen for radiosensitivity, and is associated with an adverse outcome. This reduced perfusion was found in larger, more advanced stage tumors, and is indicative for low microvessel density, low velocity and hypoxia, due to the incompetent microvessels and increased interstitial pressure [16, 18, 37, 38]. A high/or increased metabolism was also associated with adverse outcome, which might be due to a high/increased glucose demand of advanced staged tumors [39], due to proliferating malignant cells and stromal tissue. In contrast, reduced metabolism in the tumoral center due to diminished access of nutrition and oxygen supply, leading to necrosis with hypoxia, was also associated with adverse outcome [40]. These tumor characteristics might be used to target subvolumes for dose-paint RT [2, 3, 15].

### Recurrence-free survival

In the present study, the combination of HPV status, tumor volume (ADC_GTV_), high K^trans^, and high V_e_ showed more predictive potential for locoregional control than the clinically used risk stratification per T-stage. The more of these adverse factors, the worse the locoregional-free survival was. The previously described predictive value of K^trans^ [21, 23] and V_e_ was confirmed in this study [22, 23]. In contrast, Ng et al [41] found K_ep_, SUV_max_, TLG, and LNM V_e_, and ADC_mean_ as predictive parameters for LRFS. However, their chemotherapy scheme was uncommon, delineation was performed on the single-slice largest diameter, and HPV status was not assessed. In this study, ADC_mean_ was not found to be predictive for LRFS, which was also confirmed by King et al [19]. However, in small studies (*N* = 17 patients, [42], *N* = 40, [43], *N* = 32, [18]) with single modality predictive assessment, a low ADC_mean_ was found predictive for LRFS. Furthermore, we found that ^18^F-FDG-PET/CT parameters did not remain predictive when combining modalities, which was in line with Ng et al [22]. However, in single modality studies [9, 23], SUV parameters were found predictive for LRFS. Such discrepancies in predictive value may be explained by factors such as sample sizes, treatment protocols, and multivariable Cox regression analysis with or without inclusion of important clinical parameters [22].

### Overall survival

The combination of all modalities showed that N-stage, HPV-negative status, hypopharyngeal PT location, and intoxication were risk factors for adverse overall survival. This was in line with other studies who found hypopharyngeal PT location [44], alcohol use [23], and HPV status [24] as predictors.

Besides, a large tumor volume (ADC_GTV_, DCE_GTV_, MATV), K^trans^, and V_e_ (as were predictive for adverse LRFS), also high ADC_mean_, D*, SUV_max_, and TLG, were predictive for adverse OS. The more of these adverse factors, the worse the overall survival was. Previous studies performing multivariable analysis were in line with these findings and found that V_e_ [22] and K^trans^ were predictive for OS. The pretreatment finding of low ADC_mean_ was associated with highly cellular tumors including rapidly dividing cells, which are more sensitive to subsequent chemotherapy and radiotherapy and therefore associated with a more favorable prognosis [18, 19]. A possible explanation for the extra predictors for OS compared with the predictors of LRFS is that certain tumor tissue architecture, e.g., heterogeneous tissue with low diffusion restriction (high ADC_mean_) and aggressive high metabolism (high SUV_max_ and TLG), is less sensitive to (chemo)radiotherapy, which additionally decreases tumor control and survival.

In previous studies, smoking, K^trans^, K_ep_ [23, 41], and a heterogeneity ^18^F-FDG-PET/CT parameter (18F-FDG-PET uniformity) were reported to be predictive for OS. In contrast, the significant predictive value of the various clinical parameters combined with significant parameters from the whole spectrum of DWI with IVIM, DCE, and ^18^F-FDG-PET/CT was not described previously. The aforementioned risk factors for adverse OS were found significantly predictive, whereas stratification per T-stage was not significantly predictive. This implies an additional predictive value of functional imaging to clinical staging based on morphology.

### Complementarity and applicability

In order to improve prediction accuracy, the complementary value of each imaging modality is of importance to capture the whole spectrum of predictive tumoral characteristics, such as tumoral cellularity, necrosis, vascularity, and metabolism [11–13, 16, 24]. Previously described hypothetical overlapping parameters, such as DWI, IVIM (e.g., ADC_mean_ and *D*, both capturing tissue cellularity indirectly), and DCE (e.g., K^trans^ with K_ep_) [11, 12, 45], correlated not evidently in this study. Different heterogeneous tumor architecture (inflammation, fibrosis, necrosis, and hypoxia) and/or HPV status in more advanced staged tumors might have caused the loss of correlations. Further optimization of protocols and selection and evaluation of qualitative and quantitative parameters is necessary in future studies.

The current study underlines the superiority of combining MRI and ^18^F-FDG-PET/CT, which allowed combining significant predictive clinical parameters, such as HPV-negative status, intoxication (smoking/alcohol), hypopharyngeal tumor location, and N-stage with predictive quantitative imaging parameters: ADC_mean_ (DWI) and D* (IVIM), K^trans^, V_e_ (DCE) and SUV_max_, and TLG (^18^F-FDG-PET/CT) for OS. In this way, risk stratification on a patient level was shown to be possible. Furthermore, this might improve patient care and pave the road for personalized treatment options by identification and targeting tumoral subvolumes which are predictive for adverse outcome [7, 46].

### Limitations

There was a relatively low incidence of events in our cohort; therefore, this study should be considered as hypothesis-generating. Also, selection bias might have occurred by excluding surgical treatment at the prospective selection of patients with curative (chemo)radiotherapy.

Secondly, although T-stage is dependent on the gross tumor volume, they were both included in the predictive analysis, which might have caused confounding bias. Although in this study the GTV was determined on functional imaging maps (ADC and DCE maps), it should be evaluated in future studies whether GTV determined on anatomical MRI sequences is more accurate in the predictive analyses.

Thirdly, we performed pharmacokinetic modeling analysis by using a patient-specific AIF, measured in the external carotid arteries. Flow artifacts as well as high concentrations of contrast agent can result in incorrect amplitude of the arterial concentration. This can affect final calculation of K^trans^ and V_e_ which, as a consequence, are over-estimated (e.g., V_e_ can be larger than 1). We have decided to leave the results as we obtained them, but in future studies, we intend to correct the AIF for flow artifacts.

Finally, the LNM parameters were based on the ROIs of the largest lymph node metastasis, which might falsely ignore the adverse effect of having multiple metastases and consisting of necrotic tumoral areas, which reduced the average tumoral FDG uptake. The LNM parameters were excluded in the multimodality CoxBoost analysis in order to remain statistically robust, which might have limited the predictive value. Moreover, only internal validation by bootstrap cross-validation was feasible. These limitations were managed by performing a well setup internal validation by bootstrap cross-validation to test limited parameters repeatedly in a subset.

## Conclusion

The combination of clinical parameters, HPV status, with DCE, IVIM-MRI, and ^18^F-FDG-PET/CT, provided complementary value in capturing tumor characteristics and improved prediction of locoregional recurrence-free survival and overall survival. HPV-negative status, intoxications, high tumor volume, and permeability and extravascular extracellular space on DCE imaging were predictive for locoregional recurrence and decreased overall survival. Additionally, low cellularity on the ADC map and high metabolism on the ^18^F-FDG-PET/CT were additionally predictive for adverse overall survival.

## Electronic supplementary material

ESM 1 (DOCX 76 kb)

## Abbreviations

AIF: Arterial input function
AJCC: American Joint Committee on Cancer
D: Pure diffusion coefficient
D*: Pseudo-diffusion coefficient
f: Perfusion fraction
GTV: Gross tumor volume
IVIM: Intravoxel incoherent motion
K_ep_: The rate constant for transfer of contrast agent from extravascular, extracellular space to the plasma
K^trans^: The rate constant for transfer of contrast agent from plasma to extravascular, extracellular space
V_e_: The fractional volume of extravascular extracellular space
