# Supplementary material for: Multiparametric functional MRI and 18F-FDG-PET for survival prediction in patients with head and neck squamous cell carcinoma treated with (chemo)radiation
Source: Eur Radiol. 2020 Aug 26;31(2):616–28. doi: 10.1007/s00330-020-07163-3 (PMC7813703; doi:10.1007/s00330-020-07163-3)
Supplement: Supplementary file 1 — (DOCX 76 kb) [file 330_2020_7163_MOESM1_ESM.docx]

**Supplement 1.**

|  |  | Primary tumor | | | | Lymph node metastasis | | | |
| --- | --- | --- | --- | --- | --- | --- | --- | --- | --- |
|  |  | Observer 1 | Observer 2 | Difference | Correlation | Observer 1 | Observer 2 | Difference | Correlation |
|  |  | Mean (SD) | Mean (SD) | Wilcoxon Signed rank test | Pearsons r | Mean (SD) | Mean (SD) | Wilcoxon Signed rank test | Pearsons r |
| DWI | ADC volume | 9237.39 | 8994.05 | 0.08 | 0.989 | 6339.71 | 6423.85 | 0.79 | 0.978 |
|  | ADC mean | 1.22 (0.34) | 1.21 (0.33) | 0.26 | 0.988 | 1.19 (0.29) | 1.2 (0.29) | 0.70 | 0.990 |
| IVIM | D | 0.97 (0.27) | 0.97 (0.26) | 0.13 | 0.989 | 0.99 (0.26) | 1 (0.26) | 0.47 | 0.982 |
|  | D* | 17.66 (21.82) | 17.59 (21.76) | 0.19 | 0.982 | 16.72 (20.29) | 16.2 (19.84) | 0.64 | 0.947 |
|  | f | 143.43 (107.05) | 142.96 (106.04) | 0.63 | 0.986 | 115.95 (89.35) | 115.28 (88.23) | 0.81 | 0.988 |
| DCE | DCE volume | 12114.73 | 10841.81 | 0.07 | 0.597 | 4889.81 | 5033.31 | 0.09 | 0.925 |
|  | Ktrans | 355.82 (217.25) | 367.30 (211.29) | 0.33 | 0.897 | 391.63 (233.19) | 405.65 (234.52) | 0.69 | 0.989 |
|  | KEP | 1094.67 (836.79) | 1108.25 (811.85) | 0.41 | 0.743 | 1045.17 (820.92) | 1063.45 (822.22) | 0.29 | 0.998 |
|  | Ve | 176.05 (168.57) | 178.42 (169.81) | 0.10 | 0.843 | 188.57 (184.79) | 193.43 (183.19) | 0.17 | 0.844 |
|  | Vp | 97.48 (47.33) | 94.88 (48.91) | 0.15 | 0.835 | 93.94 (50.37) | 94.13 (48.33) | 0.31 | 0.994 |

**Supplement 1. The mean, standard deviation (SD), the Wilcoxon signed rank test and the Pearson interobserver correlation test, of all quantitative MRI-parameters (DWI, IVIM and DCE) calculated for the two observers. Overall, there were found no significant differences between the delineations of both observers and the correlation ranged from excellent (r > 0.8) to good (0.6 < r < 0.8), except for DCE volume (r=0.597).**

**Supplement 2. Imaging parameters per subgroup in primary tumors**

| Primary tumor | | | | | | | | | | | | | | | | |
| --- | --- | --- | --- | --- | --- | --- | --- | --- | --- | --- | --- | --- | --- | --- | --- | --- |
|  | Parameters  (mean±stddev) | T-stage | | | | N-stage | | | | HPV-status | | | Intoxications | | | |
|  |  | T2 | T3 | T4 | P-value* | N0 | N1 | N2 | P-value* | Positive | Negative | P-value** | None | Alcohol/smoking | Both | P-value |
| DWI | ADC_GTV_ (x10^3^ cm^3^) | 0.33±0.6 | 0.37±0.6 | 1.36±1.1 | 0.000004 | 0.32±0.5 | 0.49±0.7 | 0.86±1.1 | 0.021 | 0.83±0.7 | 1.03±1.2 | 0.934 | 0.47±0.7 | 0.80±0.6 | 0.49±1.4 | 0.678 |
|  | ADC_mean_  (x10^3^ mm2/s) | 1.14±0.2 | 1.26±0.2 | 1.19±0.2 | 0.095 | 1.22±0.1 | 1.19±0.2 | 1.18±0.2 | 0.839 | 1.11±0.1 | 1.28±0.2 | 0.0001 | 1.10±0.1 | 1.24±0.1 | 1.21±0.2 | 0.003 |
| IVIM | D (mm^2^/s) | 0.84±0.2 | 1.03±0.2 | 0.96±0.2 | 0.010 | 1.03±0.2 | 0.92±0.2 | 0.92±0.2 | 0.486 | 0.85±0.1 | 1.03±0.2 | 0.0001 | 0.85±0.1 | 1.02±0.2 | 0.96±0.2 | 0.006 |
|  | f (mm^2^/s) | 0.16±0.1 | 0.13±0.1 | 0.11±0.0 | 0.002 | 1.09±0.5 | 0.15±0.5 | 1.24±0.6 | 0.268 | 1.30±0.4 | 1.46±0.5 | 0.427 | 1.34±0.4 | 1.24±0.5 | 1.26±0.6 | 0.701 |
|  | D* (mm^2^/s) | 0.21±0.1 | 0.15±0.1 | 0.15±0.1 | 0.021 | 0.16±0.1 | 0.22±0.1 | 0.16±0.1 | 0.072 | 0.20±0.1 | 0.16±0.1 | 0.004 | 0.21±0.1 | 0.14±0.1 | 0.18±0.1 | 0.027 |
| DCE | DCE_GTV_ (cm^3^) | 4.55±6.4 | 7.97±9.0 | 18.0±13.6 | 0.000 | 5.11±5.6 | 8.63±8.3 | 10.86±13.6 | 0.115 | 10.7±8.2 | 14.8±15.3 | 0.549 | 7.38±9.4 | 12.65±13.1 | 9.50±14.5 | 0.559 |
|  | K_ep_  (min^−1^) | 1.05±0.3 | 1.15±0.6 | 1.19±0.4 | 0.148 | 1.01±0.4 | 1.08±0.4 | 1.12±0.5 | 0.561 | 1.19±0.4 | 1.12±0.5 | 0.428 | 0.879±0.3 | 0.782±0.3 | 0.76±0.5 | 0.811 |
|  | K^trans^ (min^−1^) | 0.53±0.2 | 0.48±0.4 | 0.64±0.3 | 0.066 | 0.45±0.5 | 0.53±0.3 | 0.60±0.3 | 0.322 | 0.63±0.3 | 0.62±0.3 | 0.495 | 0.708±1.5 | 1.02±1.1 | 1.01±1.6 | 0.979 |
|  | V_e_ | 0.99±0.9 | 1.14±0.6 | 1.04±1.5 | 0.311 | 0.63±0.6 | 1.03±1.0 | 1.15±1.2 | 0.023 | 1.27±0.7 | 1.42±1.1 | 0.963 | 0.87±1.2 | 1.22±0.9 | 1.07±1.5 | 0.702 |
| ^18^F-FDG-PET | MATV (cm^3^) | 4.45±5.0 | 7.97±3.9 | 14.0±14.0 | .00008 | 7.62±4.9 | 6.3±6.4 | 8.2±12.3 | 0.431 | 8.9±6.5 | 12.4±13.0 | 0.297 | 6.3±6.0 | 9.0±6.6 | 8.7±15.0 | 0.302 |
|  | SUV_max_ (Bq) | 6.9±2.0 | 10.2±5.7 | 11.0±4.0 | .00003 | 9.97±4.5 | 7.5±3.4 | 8.7±4.8 | 0.593 | 7.4±1.8 | 10.4±5.0 | 0.021 | 7.50±5.3 | 10.4±4.0 | 8.4±3.7 | 0.256 |
|  | SUV_mean_ (Bq) | 4.1±1.3 | 6.3±3.7 | 6.6±2.3 | .00003 | 5.94±2.1 | 5.2±2.2 | 5.7±3.1 | 0.595 | 5.0±1.3 | 6.7±3.3 | 0.033 | 5.3±3.4 | 6.3±2.3 | 5.3±2.8 | 0.288 |
|  | SUV_peak_ (Bq) | 4.9±1.7 | 7.78±4.9 | 8.5±2.9 | .00001 | 7.4±2.6 | 6.25±2.9 | 7.0±4.1 | 0.696 | 6.1±1.7 | 8.3±4.3 | 0.043 | 6.4±4.5 | 7.8±3.0 | 6.4±3.1 | 0.307 |
|  | TLG (Bq*cm^3^) | 0.20±0.4 | 0.48±0.6 | 0.78±0.9 | .00000 | 0.44±0.3 | 0.31±0.6 | 0.46±0.9 | 0.437 | 0.48±0.5 | 0.9±0.9 | 0.112 | 0.29±0.7 | 0.59±58.3 | 0.41±01.3 | 0.556 |
|  | * | Kruskal-Wallis Test | |  |  |  |  |  |  |  |  |  |  |  |  |  |
|  | ** | Mann-Whitney U | |  |  |  |  |  |  |  |  |  |  |  |  |  |
|  | † | Smoking:>20packyears | |  |  |  |  |  |  |  |  |  |  |  |  |  |

**Supplement 2.** The mean of the primary tumor imaging parameters per T-stage, N-stage, HPV-status and intoxications. The significant differences between these groups is marked green (p<0.05).

**Supplement 3 – Imaging parameters per subgroup in lymph node metastases**

| Lymph node metastases | | | | | | | | | | | | | | | | |
| --- | --- | --- | --- | --- | --- | --- | --- | --- | --- | --- | --- | --- | --- | --- | --- | --- |
|  | Parameters  (mean ± stddev) | T-stage | | | | N-stage | | | | HPV-status | | | Intoxications | | | |
|  |  | T2 | T3 | T4 | P-value* | N0 | N1 | N2 | P-value** | Positive | Negative | P-value** | None | Alcohol/smoking | Both | P-value |
| DWI | ADC_GTV_ (cm^3^) | 5.5±4.5 | 2.9±5.3 | 4.5±5.2 | 0.719 | - | 4.4±3.6 | 5.1±5.2 | 0.338 | 6.3±3.4 | 6.5±6.1 | 0.640 | 5.8±4.5 | 4.5±3.1 | 6.6±6.0 | 0.167 |
|  | ADC (x10^3^ mm2/s) | 1.24±0.2 | 1.03±0.3 | 1.14±0.3 | 0.747 | - | 1.2±2.4 | 1.2±0.3 | 0.903 | 1.1±0.2 | 1.2±0.3 | 0.089 | 1.2±0.2 | 1.2±0.3 | 1.1±0.3 | 0.999 |
| IVIM | D (mm^2^/s) | 0.79±0.2 | 0.72±0.2 | 0.82±0.2 | 0.819 | - | 0.75±0.2 | 0.82±0.2 | 0.210 | 0.7±0.2 | 0.9±0.2 | 0.002 | 0.8±0.2 | 0.7±0.2 | 0.9±0.3 | 0.306 |
|  | F (mm^2^/s) | 1.9±0.6 | 1.79±0.6 | 1.74±0.7 | 0.256 | - | 2.0±0.7 | 1.71±0.6 | 0.085 | 1.9±0.6 | 1.8±0.6 | 0.442 | 2.1±0.5 | 1.8±0.8 | 1.5±0.4 | 0.026 |
|  | D* (mm^2^/s) | 2.6±0.7 | 2.6±0.6 | 2.5±0.7 | 0.529 | - | 2.87±0.8 | 2.49±0.6 | 0.180 | 2.7±0.6 | 2.2±0.5 | 0.007 | 2.6±0.6 | 2.6±0.7 | 2.2±0.7 | 0.205 |
| DCE | DCE_GTV_ (cm^3^) | 6.2±4.1 | 4.4±4.2 | 3.3±3.1 | 0.123 | - | 4.1±4.4 | 4.4±3.8 | 0.352 | 5.6±3.8 | 4.9±4.0 | 0.403 | 4.6±4.4 | 3.6±3.8 | 5.9±3.5 | 0.577 |
|  | K_ep_ (min^−1^) | 0.80±0.6 | 0.59±0.7 | 0.87±0.5 | 0.519 | - | 1.03±0.6 | 0.75±0.6 | 0.025 | 1.0±0.6 | 0.9±0.6 | 0.144 | 1.0±0.7 | 0.7±0.5 | 0.8±0.5 | 0.051 |
|  | K^trans^ (min^−1^) | 0.55±1.2 | 0.79±1.3 | 1.28±1.1 | 0.121 | - | 0.72±1.3 | 0.78±1.2 | 0.347 | 1.3±1.1 | 1.2±1.2 | 0.350 | 0.9±1.1 | 1.0±1.0 | 0.6±1.4 | 0.323 |
|  | VE | 0.83±0.7 | 1.01±0.8 | 1.14±1.3 | 0.317 | - | 0.95±0.7 | 1.04±1.1 | 0.903 | 1.4±0.8 | 1.4±1.2 | 0.271 | 1.0±0.8 | 1.1±0.8 | 1.0±1.4 | 0.893 |
| ^18^F-FDG-PET | MATV (cm^3^) | 4.0±3.4 | 4.0±9.3 | 5.2±7.6 | 0.798 | - | 2.7±2.8 | 6.0±7.5 | 0.011 | 5.1±3.3 | 6.6±7.4 | 0.907 | 3.2±3.6 | 4.2±8.8 | 4.0±8.4 | 0.614 |
|  | SUV_max_ (Bq) | 7.8±2.8 | 9.7±4.6 | 7.3±4.8 | 0.718 | - | 3.9±1.5 | 5.7±2.3 | 0.007 | 6.8±2.8 | 8.4±4.2 | 0.164 | 4.8±1.7 | 4.5±2.9 | 6.1±2.2 | 0.316 |
|  | SUV_mean_ (Bq) | 4.66±1.7 | 6.31±2.6 | 4.3±2.6 | 0.706 | - | 5.8±2.7 | 8.8±4.1 | 0.007 | 4.4±1.7 | 5.3±2.4 | 0.170 | 8.2±2.8 | 6.9±5.6 | 9.4±3.8 | 0.274 |
|  | SUV_peak_ (Bq) | 5.6±2.4 | 7.9±3.7 | 4.9±3.5 | 0.720 | - | 4.4±2.1 | 6.4±3.2 | 0.016 | 5.3±2.3 | 6.4±3.5 | 0.176 | 6.1±2.2 | 5.4±4.2 | 7.3±3.1 | 0.297 |
|  | TLG (Bq*cm^3^) | 3.2±8.3 | 4.4±17.5 | 3.7±11.2 | 0.653 | - | 1.6±3.2 | 4.4±13.2 | 0.008 | 5.0±5.7 | 8.6±11.6 | 0.397 | 2.3±3.3 | 3.5±16.5 | 3.9±6.6 | 0.469 |
|  | * | Kruskal-Wallis Test | |  |  |  |  |  |  |  |  |  |  |  |  |  |
|  | ** | Mann-Whitney U | |  |  |  |  |  |  |  |  |  |  |  |  |  |

**Supplement 3.** The mean of the lymph node metastasis imaging parameters per T-stage, N-stage, HPV-status and intoxications. The significant differences between these groups are marked green (p<0.05).

**Supplement 4. Correlations between imaging parameters extracted from the primary tumor.**

|  | Primary tumor | | | | | | | | |
| --- | --- | --- | --- | --- | --- | --- | --- | --- | --- |
|  | K^trans^ | K_ep_ | V_e_ | DCE_GTV_ | SUV_max_ | SUV_peak_ | SUV_mean_ | TLG | MATV |
| ADC_mean_ | 0.121 | 0.095 | 0.104 | -0.026 | 0.124 | 0.105 | 0.117 | 0.023 | 0.005 |
| p-value | 0.112 | 0.193 | 0.233 | 0.797 | 0.311 | 0.391 | 0.338 | 0.851 | 0.965 |
| D | 0.240 | 0.120 | 0.117 | 0.148 | 0.286 | 0.260 | 0.285 | 0.127 | 0.040 |
| p-value | 0.052 | 0.337 | 0.349 | 0.236 | 0.017 | 0.031 | 0.018 | 0.298 | 0.746 |
| D* | -0.247 | -0.049 | -0.098 | -0.256 | -0.267 | -0.294 | -0.281 | -0.276 | -0.261 |
| p-value | 0.046 | 0.697 | 0.432 | 0.038 | 0.027 | 0.014 | 0.019 | 0.022 | 0.031 |
| f | -0.186 | -0.086 | -0.047 | -0.318 | -0.232 | -0.266 | -0.258 | -0.300 | -0.257 |
| p-value | 0.134 | 0.492 | 0.710 | 0.009 | 0.055 | 0.027 | 0.033 | 0.012 | 0.033 |
| ADC_GTV_ | 0.164 | 0.151 | 0.250 | **0.872** | 0.358 | **0.434** | **0.392** | **0.869** | **0.915** |
| p-value | 0.189 | 0.226 | 0.043 | 0.000 | 0.003 | 0.000 | 0.001 | 0.000 | 0.000 |
| SUV_max_ | 0.107 | 0.098 | 0.151 | **0.442** |  |  |  |  |  |
| p-value | 0.397 | 0.435 | 0.229 | 0.000 |  |  |  |  |  |
| SUV_mean_ | 0.133 | 0.108 | 0.157 | **0.469** |  |  |  |  |  |
| p-value | 0.291 | 0.390 | 0.212 | 0.000 |  |  |  |  |  |
| SUV_peak_ | 0.155 | 0.130 | 0.157 | **0.509** |  |  |  |  |  |
| p-value | 0.218 | 0.301 | 0.212 | 0.000 |  |  |  |  |  |
| TLG | 0.210 | 0.188 | 0.257 | **0.827** |  |  |  |  |  |
| p-value | 0.093 | 0.134 | 0.039 | 0.000 |  |  |  |  |  |
| MATV | 0.196 | 0.188 | 0.245 | **0.830** |  |  |  |  |  |
| p-value | 0.117 | 0.134 | 0.049 | 0.000 |  |  |  |  |  |

**Supplement 4**. Correlation (Pearson test) between functional imaging parameters of different modalities. Significant correlations after Bonferroni’s correction (p=0.002) are bold.

**Supplement 5. Correlations between imaging parameters extracted from the lymph node metastasis.**

|  | Lymph node metastasis | | | | | | | | |
| --- | --- | --- | --- | --- | --- | --- | --- | --- | --- |
|  | K^trans^ | K_ep_ | V_e_ | DCE_GTV_ | SUV_max_ | SUV_peak_ | SUV_mean_ | TLG | MATV |
| ADC_mean_ | 0.107 | 0.053 | 0.096 | 0.052 | 0.02 | 0.086 | 0.046 | 0.009 | 0.137 |
| p-value | 0.418 | 0.688 | 0.467 | 0.697 | 0.881 | 0.516 | 0.727 | 0.946 | 0.299 |
| D | 0.030 | -0.098 | 0.148 | 0.037 | 0.115 | 0.184 | 0.150 | 0.169 | 0.256 |
| p-value | 0.821 | 0.459 | 0.264 | 0.781 | 0.387 | 0.162 | 0.257 | 0.200 | 0.051 |
| D* | 0.019 | 0.085 | -0.145 | -0.049 | -0.127 | -0.151 | -0.135 | -0.151 | -0.112 |
| p-value | 0.884 | 0.521 | 0.273 | 0.711 | 0.339 | 0.253 | 0.309 | 0.255 | 0.398 |
| f | 0.119 | 0.174 | -0.049 | 0.036 | -0.094 | -0.090 | -0.091 | -0.223 | -0.104 |
| p-value | 0.368 | 0.188 | 0.710 | 0.785 | 0.478 | 0.499 | 0.494 | 0.090 | 0.432 |
| ADC_GTV_ | -0.241 | -0.204 | -0.082 | **0.725** | 0.190 | 0.249 | 0.187 | 0.344 | **0.651** |
| p-value | 0.066 | 0.120 | 0.538 | 0.000 | 0.150 | 0.058 | 0.156 | 0.008 | 0.000 |
| SUV_max_ | -0.204 | -0.048 | -0.150 | 0.247 |  |  |  |  |  |
| p-value | 0.122 | 0.718 | 0.257 | 0.059 |  |  |  |  |  |
| SUV_mean_ | -0.189 | -0.056 | -0.117 | 0.306 |  |  |  |  |  |
| p-value | 0.152 | 0.673 | 0.376 | 0.018 |  |  |  |  |  |
| SUV_peak_ | -0.197 | -0.063 | -0.112 | 0.237 |  |  |  |  |  |
| p-value | 0.136 | 0.636 | 0.398 | 0.070 |  |  |  |  |  |
| TLG | -0.126 | -0.181 | -0.072 | 0.266 |  |  |  |  |  |
| p-value | 0.341 | 0.169 | 0.589 | 0.041 |  |  |  |  |  |
| MATV | -0.050 | -0.186 | 0.081 | **0.399** |  |  |  |  |  |
| p-value | 0.707 | 0.158 | 0.540 | 0.002 |  |  |  |  |  |

**Supplement 5**. Correlation (Pearson test) between functional imaging parameters of different modalities. Significant correlations after Bonferroni’s correction (p=0.002) are bold.

**Supplement 6.**

| **A** | Primary tumor | | | | |  | **B** | Primary tumor | | | |  | **C** | Primary tumor | | | |  |
| --- | --- | --- | --- | --- | --- | --- | --- | --- | --- | --- | --- | --- | --- | --- | --- | --- | --- | --- |
|  | ADC_mean_ | D | D* | f | ADCGTV |  |  | K^trans^ | K_ep_ | V_e_ | DCE_GTV_ |  |  | SUV_max_ | SUV_peak_ | SUV_mean_ | TLG | MATV |
| ADC_mean_ |  |  |  |  |  |  | K^trans^ |  |  |  |  |  | SUV_max_ |  |  |  |  |  |
| p-value |  |  |  |  |  |  | p-value |  |  |  |  |  | p-value |  |  |  |  |  |
| D | **0.670** |  |  |  |  |  | K_ep_ | **0.711** |  |  |  |  | SUV_peak_ | **0.980** |  |  |  |  |
| p-value | 0.000 |  |  |  |  |  | p-value | 0.000 |  |  |  |  | p-value | 0.000 |  |  |  |  |
| D* | -0.205 | **-0.501** |  |  |  |  | V_e_ | 0.338 | 0.068 |  |  |  | SUV_mean_ | **0.982** | **0.995** |  |  |  |
| p-value | 0.089 | 0.000 |  |  |  |  | p-value | 0.005 | 0.59 |  |  |  | p-value | 0.000 | 0.000 |  |  |  |
| f | 0.292 | -0.277 | **0.563** |  |  |  | DCE_GTV_ | 0.161 | 0.143 | 0.231 |  |  | TLG | **0.582** | **0.640** | **0.612** |  |  |
| p-value | 0.014 | 0.020 | 0.000 |  |  |  | p-value | 0.196 | 0.251 | 0.062 |  |  | p-value | 0.000 | 0.000 | 0.000 |  |  |
| ADC_GTV_ | -0.015 | 0.053 | -0.267 | -0.342 |  |  |  |  |  |  |  |  | MATV | 0.236 | 0.300 | 0.257 | **0.897** |  |
| p-value | 0.899 | 0.663 | 0.026 | 0.004 |  |  |  |  |  |  |  |  | p-value | 0.051 | 0.012 | 0.033 | 0.000 |  |
| **D** | Lymph node metastasis | | | | |  | **E** | Lymph node metastasis | | | |  | **F** | Lymph node metastasis | | | |  |
|  | ADC_mean_ | D | D* | f | ADC_GTV_ |  |  | K^trans^ | K_ep_ | V_e_ | DCE_GTV_ |  |  | SUV_max_ | SUV_peak_ | SUV_mean_ | TLG | MATV |
| ADC_mean_ |  |  |  |  |  |  | K^trans^ |  |  |  |  |  | SUV_max_ |  |  |  |  |  |
| p-value |  |  |  |  |  |  | p-value |  |  |  |  |  | p-value |  |  |  |  |  |
| D | **0.794** |  |  |  |  |  | K_ep_ | **0.637** |  |  |  |  | SUV_peak_ | 0.270 |  |  |  |  |
| p-value | 0.000 |  |  |  |  |  | p-value | 0.000 |  |  |  |  | p-value | 0.039 |  |  |  |  |
| D* | -0.335 | **-0.670** |  |  |  |  | V_e_ | **0.741** | 0.081 |  |  |  | SUV_mean_ | 0.260 | **0.978** |  |  |  |
| p-value | 0.010 | 0.000 |  |  |  |  | p-value | 0.000 | 0.541 |  |  |  | p-value | 0.047 | 0.000 |  |  |  |
| f | **0.494** | -0.096 | **0.432** |  |  |  | DCE_GTV_ | -0.286 | -0.112 | -0.221 |  |  | TLG | 0.344 | **0.981** | **0.978** |  |  |
| p-value | 0.000 | 0.470 | 0.001 |  |  |  | p-value | 0.028 | 0.400 | 0.092 |  |  | p-value | 0.008 | 0.000 | 0.000 |  |  |
| ADC_GTV_ | 0.144 | 0.198 | -0.118 | -0.015 |  |  |  |  |  |  |  |  | MATV | **0.698** | 0.363 | 0.351 | **0.409** |  |
| p-value | 0.278 | 0.133 | 0.373 | 0.910 |  |  |  |  |  |  |  |  | p-value | 0.000 | 0.005 | 0.006 | 0.001 |  |

**Supplement 6**. Correlation (Pearson test) between functional imaging parameters of similar modalities. Significant correlations after Bonferroni’s correction (p=0.002) are bold.

**Supplement7.**

| HPV-negative tumors | | | | | | | |  |  |
| --- | --- | --- | --- | --- | --- | --- | --- | --- | --- |
| n=44 patients  Parameters | | | Multivariable  Recurrence | | Multivariable overall survival | | |  |  |
|  |  |  | p-value* | HR (95%CI) | p-value* | HR (95%CI) | |  |  |
| Clinical parameters | | Gender | - |  | - |  | |  |  |
|  |  | Age | - |  | - |  | |  |  |
|  |  | T-stage | - |  | - |  | |  |  |
|  |  | N-stage | - |  | - |  | |  |  |
|  |  | HPV | - |  | - |  | |  |  |
|  |  | Location PT | - |  | - |  | |  |  |
|  |  | Smoking (PY) | - |  | - |  | |  |  |
|  |  | Alcohol (≥3drinks/day) | - |  | - |  | |  |  |
|  |  | Intoxications | - |  | - |  | |  |  |
|  |  | None | - |  | - |  | |  |  |
|  |  | Smoking or alcohol use | - |  | - |  | |  |  |
|  |  | Smoking and alcohol use | - |  | - |  | |  |  |
| Primary tumor | DWI | ADCGTV | 0.009 | 1.84 (1.17-2.90) | 0.002 | 1.82 (1.2-2.7) | |  |  |
|  |  | ADC | - |  | - |  | |  |  |
|  | IVIM | D* | - |  | - |  | |  |  |
|  |  | D | - |  | - |  | |  |  |
|  |  | f | - |  | - |  | |  |  |
|  | DCE | DCEGTV | 0.086 | 1.03 (1.0-1.1) | 0.037 | 1.03 (1.0-1.1) | |  |  |
|  |  | K_ep_ | 0.012 | 4.32 (1.4-13.5) | 0.054 | 2.80 (1.0-8.0) | |  |  |
|  |  | K^trans^ | - |  | - |  | |  |  |
|  |  | V_e_ | 0.053 | 1.50 (1.0-2.3) | 0.051 | 1.40 (1.0-2.0) | |  |  |
|  |  | MATV | 0.029 | 1.05 (1.0-1.1) | - |  | |  |  |
|  | ^18^F-FDG-PET | SUV_max_ | - |  | - |  | |  |  |
|  |  | SUV_mean_ | - |  | - |  | |  |  |
|  |  | SUV_peak_ | - |  | - |  | |  |  |
|  |  | TLG | - |  | 0.001 | 1.01 (1.0-1.01) | |  |  |
| Lymphnode metastases | DWI | ADC_GTV_ (*10e3) | - |  | - |  | |  |  |
|  |  | ADC | - |  | - |  | |  |  |
|  | IVIM | D* | - |  | - |  | |  |  |
|  |  | D | - |  | - |  | |  |  |
|  |  | f | - |  | - |  | |  |  |
|  | DCE | DCEGTV | 0.027 | 1.20 (1.0-1.4) | - |  | |  |  |
|  |  | K_ep_ | - |  | - |  | |  |  |
|  |  | K^trans^ | - |  | - |  | |  |  |
|  |  | V_e_ | - |  | - |  | |  |  |
|  | ^18^F-FDG-PET | MATV | - |  | - |  | |  |  |
|  |  | SUV_max_ | 0.077 | 0.45 (0.2-1.1) | - |  | |  |  |
|  |  | SUV_mean_ | - |  | 0.08 | 1.19 (1.0-1.5) | |  |  |
|  |  | SUV_peak_ | 0.064 | 2.90 (0.9-9.0) | - |  | |  |  |
|  |  | TLG | - |  | - |  | |  |  |
|  | * | Multivariable Cox-regression analysis | | |  | |  | |  |

**Supplement 7**. Multivariable sub-group analysis in HPV-negative patients of functional imaging parameters per modality predicting Recurrence-free survival and overall survival. Significantly predictive parameters are marked green.
